# Insight into the Antibacterial Activity of Selected Metal Nanoparticles and Alterations within the Antioxidant Defence System in *Escherichia coli*, *Bacillus cereus* and *Staphylococcus epidermidis*

**DOI:** 10.3390/ijms222111811

**Published:** 2021-10-30

**Authors:** Oliwia Metryka, Daniel Wasilkowski, Agnieszka Mrozik

**Affiliations:** 1 Doctoral School, University of Silesia, Bankowa 14, 40-032 Katowice, Poland; 2 Institute of Biology, Biotechnology and Environmental Protection, Faculty of Natural Sciences, University of Silesia, Jagiellońska 28, 40-032 Katowice, Poland; daniel.wasilkowski@us.edu.pl

**Keywords:** bacteria, nanoparticles, toxicological parameters, reactive oxygen species, antioxidant enzymes, protein and lipid oxidation

## Abstract

The antimicrobial activity of nanoparticles (NPs) is a desirable feature of various products but can become problematic when NPs are released into different ecosystems, potentially endangering living microorganisms. Although there is an abundance of advanced studies on the toxicity and biological activity of NPs on microorganisms, the information regarding their detailed interactions with microbial cells and the induction of oxidative stress remains incomplete. Therefore, this work aimed to develop accurate oxidation stress profiles of *Escherichia coli*, *Bacillus cereus* and *Staphylococcus epidermidis* strains treated with commercial Ag-NPs, Cu-NPs, ZnO-NPs and TiO_2_-NPs. The methodology used included the following determinations: toxicological parameters, reactive oxygen species (ROS), antioxidant enzymes and dehydrogenases, reduced glutathione, oxidatively modified proteins and lipid peroxidation. The toxicological studies revealed that *E. coli* was most sensitive to NPs than *B. cereus* and *S. epidermidis*. Moreover, NPs induced the generation of specific ROS in bacterial cells, causing an increase in their concentration, which further resulted in alterations in the activity of the antioxidant defence system and protein oxidation. Significant changes in dehydrogenases activity and elevated lipid peroxidation indicated a negative effect of NPs on bacterial outer layers and respiratory activity. In general, NPs were characterised by very specific nano-bio effects, depending on their physicochemical properties and the species of microorganism.

## 1. Introduction

The dynamic development of education, technology, and market economy worldwide requires high advancement in the research of new materials that may resolve current global problems. Nanotechnology and its microscopic universe offer unlimited possibilities for contemporary science and various branches of industry. One of the benefits of modern nanotechnology is the production of nanomaterials (NMs) with antimicrobial activity and implementing them into different commercial processes and products as a defence against multidrug resistant infectious microorganisms [1,2,3]. Direct manipulation of synthesised NMs and adequate alteration of their properties can create broad-spectrum and long-term antimicrobials, which is a substantial trait, especially in the medical field. For instance, inorganic NMs based on metal and metal oxides have been utilised in biomedical implant materials, such as dental implants, through their application on the implant surfaces as nanocoatings or as an integral part of the material. Consecutively, this could prevent the origin and further development of infections in the place of implant insertion, increasing the chance of successful surgeries [4,5,6]. On the other hand, the release of such NMs into the environment is a potential threat to microbial populations that are not a target of their action. According to Bundschuh et al. [7], approximate global estimates of the accumulation of NMs in the environment indicate that the main accumulators of such structures are landfills (63–91%) and soil (8–28%). Despite significant advances in analytical methods, it is still impossible to measure the concentrations of NMs in various ecosystems and assess their environmental risk [7]. Therefore, it is urgent to fully evaluate the impact of NMs on different microbial strains to determine their safe use and prevent possible risks of their presence in ecological sinks.

Nanomaterials, including nanoparticles (NPs), have attracted much attention in the last decade due to their antimicrobial properties and non-specific targeting of treated microorganisms. The mode of action of NPs involves, among others, the disruption of the integrity of bacterial outer layers, increase in the permeabilisation of the bacterial cell membrane, destruction of intracellular structures, or decrease in bacterial viability [8,9]. However, one of the most attributed and principal mechanisms of NPs toxicity is the induction of oxidative stress in microbial cells. Metallic NPs can induce the generation of reactive oxygen species (ROS) inside cells, including superoxide radical anion (O_2_^•−^), hydroxyl radical (^•^OH), hydroperoxyl radical (HO_2_^•−^), hydrogen peroxide (H_2_O_2_), singlet oxygen (^1^O_2_) and organic radicals such as peroxyl radical (ROO^•^) or alkoxyl (RO^•^) [9,10,11]. However, it is worth emphasising that various ROS are produced naturally in biological systems as part of redox cycling, for example, O_2_^•−^ and H_2_O_2_ can be produced by the accidental release of electrons in the respiratory chain and auto-oxidation of dehydrogenases [12,13]. Similarly, ^1^O_2_ can be produced as a by-product in processes like lipid peroxidation, decomposition of organic peroxides or oxidation of O_2_ [14,15]. However, exposure of microorganisms to inorganic NPs can result in the greater formation of ROS, causing severe changes in their concentrations, engendering a synergistic unfavourable effect. It has been documented that metallic NPs as redox labile compounds can produce ROS, especially after prior irradiation, due to the formation of holes (h^+^) and electrons (e^−^) in conducting and valence bands, with strong redox properties [15,16,17]. NPs can also generate ROS without prior illumination due to oxidation-redox reactions on the surface of NPs, nanoparticle-bacterial cells and nanoparticle-oxidant/radical interactions [11,18]. Furthermore, the presence of transition metals on the surface of NPs provides additional generation of ROS through the catalysis of Fenton, Fenton-like and Haber-Weiss reactions [11,18,19,20].

ROS-induced stress in microbial cells can damage cellular structures and outer layers, including oxidative degradation of proteins and lipids [2,17]. Interestingly, each type of ROS is characterised by its specific reactivity. For example, induction of ^1^O_2_ can impair membrane integrity through the oxidation of cell membrane components and initiation of lipid peroxidation [16,18]. Contrarily, O_2_^•−^ may not be a strong oxidant; however, it is a substrate for H_2_O_2_, ^•^OH and ^1^O_2_ formation [10,13,18]. The high redox potential of ^•^OH makes it a very strong oxidant, which is nonselective as it can react with any macromolecules present in cells, including proteins, nucleic acids, carbohydrates, and lipids [13,18]. It has been stated previously that ^•^OH, O_2_^•−^ and ^1^O_2_ play a significant role in inducing oxidative stress in bacterial cells [16,18]. Although H_2_O_2_ is a long-lasting ROS, it is a very stable molecule, which contributes to the generation of oxidative stress through the synthesis of ^•^OH [13]. Microbial defence system against the harmful effect of NPs and ROS consists of catalase (CAT), peroxidase (PER) and superoxide dismutase (SOD) together with non-catalytic antioxidant reduced glutathione (GSH) [10,21,22,23]. CAT, PER and GSH neutralise H_2_O_2_, while SOD reduces O_2_^•−^ levels [10,12]. However, an excess of ROS in bacterial cells can lead to inactivation and permanent damage of proteins, including antioxidants, through their interactions with amino acid residues [12,22]. Therefore, it is important to monitor whether the microbial defence system can withstand cellular changes in response to NPs and ROS.

To date, comprehensive knowledge regarding the impact of NPs on the antioxidant defence system in bacteria has been obtained through many studies. Despite this, there is still limited information about the particular forms of radicals induced by redox labile Ag-NPs, Cu-NPs, ZnO-NPs and TiO_2_-NPs in bacterial cells. The research has mainly focused on oxidative stress and ROS generation by selected NPs after prior irradiation and has only considered the selected effects of NPs on antioxidant enzymes activity and cellular damages. Thus, it is crucial to also measure the cycling of ROS in bacterial cells without light-assisted conditions as well as to determine the activity of as many components as possible along with specific oxidation biomarkers. Therefore, this study aimed to create detailed oxidative stress profiles for *Escherichia coli*, *Bacillus cereus* and *Staphylococcus epidermidis* under Ag-NPs, Cu-NPs, ZnO-NPs and TiO_2_-NPs exposure. Particular research tasks included: (1) determining the toxicological parameters of NPs towards tested bacteria, (2) measuring the general and singular ROS concentration, (3) evaluating the activity of antioxidant enzymes and dehydrogenases, (4) measuring the reduced glutathione concentration, (5) assessing the protein and lipid peroxidation level and (6) establishing the relationships between measured parameters.

## 2. Results

### 2.1. Effects of the Tested NPs on Bacteria Viability

The determined minimum bactericidal concentration (MBC), minimum inhibitory concentration (MIC), and the half-maximal inhibitory concentration (IC_50_) indicated varied sensitivity of all tested strains to the presence of individual NPs (Table 1). For example, based on MIC and MBC values, *E. coli* was most sensitive to pure metal NPs than metal oxide materials. By comparison, *B. cereus* exhibited a different sensitivity to the tested NPs. The NPs with a similar bactericidal effect and the highest toxicity against this strain were Cu-NPs and TiO_2_-NPs, while Ag-NPs and ZnO-NPs had the lowest impact on cell viability. Subsequently, *S. epidermidis* was characterised by a similar trend in the sensitivity to metal NPs and metal oxide NPs as *E. coli*; however, based on IC_50_, it was more resistant to Ag-NPs, TiO_2_-NPs and ZnO-NPs. Comparing results obtained for gram-positive strains, it can be concluded that they reacted similarly to Ag-NPs, Cu-NPs and ZnO-NPs; however, TiO_2_-NPs aroused significant interest, with the IC_50_ being 14-times lower for *B. cereus* than *S. epidermidis*. Conclusively, the decreasing bactericidal properties of the tested NPs based on MIC values towards *E. coli* and *B. cereus* cells could be ordered as follows: TiO_2_-NPs < ZnO-NPs < Cu-NPs < Ag-NPs, and Ag-NPs < ZnO-NPs < TiO_2_-NPs < Cu-NPs, respectively. Unlike, the following order: TiO_2_-NPs < ZnO-NPs < Ag-NPs < Cu-NPs which presents the antibacterial effect of NPs towards *S. epidermidis*.

### 2.2. Generation of ROS in Bacterial Cells by NPs

**Figure 1:**
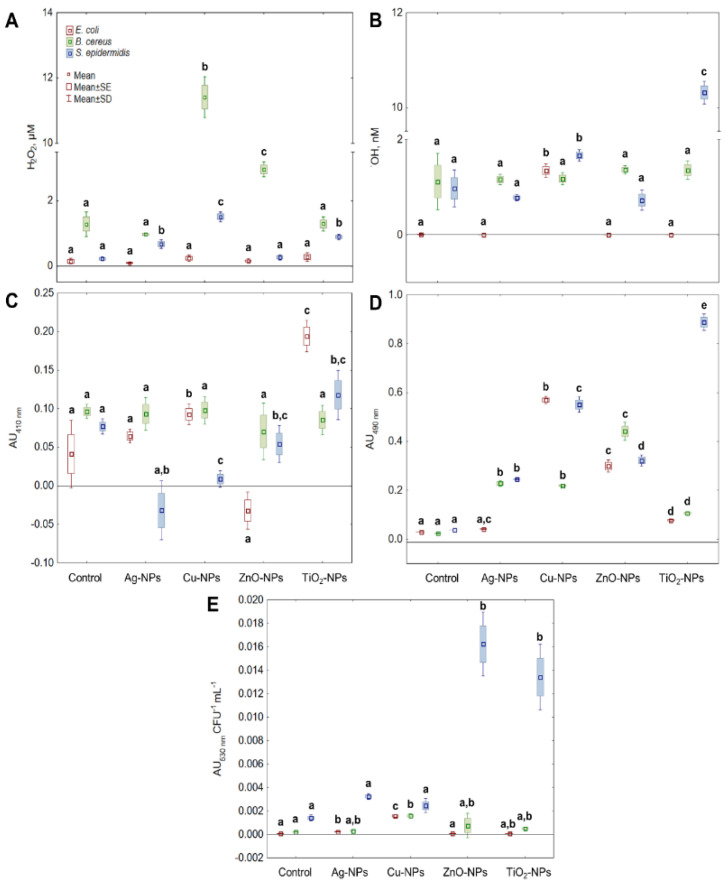
The levels of H_2_O_2_ (**A**), ^•^OH (**B**), ^1^O_2_ (**C**), O_2_^•−^ (**D**) and total ROS concentration (**E**) in *E. coli*, *B. cereus* and *S. epidermidis* exposed to NPs at an IC_50_ value (mean ± SD/SE; *n* = 3). Means with the same letter(s) are not significant at *p* < 0.05 within each ROS between the control and NPs-treated samples.

One of the studied ROS was H_2_O_2_, which is a common by-product of cell metabolic activity. It was found that the addition of Cu-NPs, ZnO-NPs, and TiO_2_-NPs into *E. coli* culture caused a negligible increase in the level of H_2_O_2_ (*p* > 0.05) (Figure 1A). In turn, the treatment of *B. cereus* with Cu-NPs and ZnO-NPs generated a considerable (*p* < 0.05) amount of H_2_O_2_, greater by about 11- and 2.7-fold compared to its content in the control sample, respectively. By comparison, Ag-NPs, Cu-NPs, and TiO_2_-NPs caused significant, for *p* < 0.05, changes in the level of H_2_O_2_ in *S. epidermidis* culture. The highest increase in this ROS concentration, about 5.5- and 3.2-fold, was recorded for Cu-NPs and TiO_2_-NPs, respectively. Overall, the treatment of bacterial cells with NPs had a more stimulating effect on the generation of H_2_O_2_ in both *B. cereus* and *S. epidermidis* than in *E. coli*.

Due to its reactivity, ^•^OH radical is considered one of the most lethal types of ROS. It is responsible for most of the damage during oxidative stress, so it was crucial to determine its level in the cells treated with NPs. It was established that the exposure of *E. coli* to Cu-NPs resulted in a vast increase in ^•^OH level compared to the control sample (Figure 1B). However, Ag-NPs, ZnO-NPs and TiO_2_-NPs did not induce substantial changes in the level of ^•^OH in these bacteria. By comparison, results obtained for *B. cereus* showed no significant differences in ^•^OH concentration between the samples treated with Ag-NPs, Cu-NPs, ZnO-NPs and TiO_2_-NPs, and the control sample. In the case of *S. epidermidis* cultures exposed to Ag-NPs and ZnO-NPs, no increase in ^•^OH levels was documented. Despite that, substantial intensification of ^•^OH generation was found in *S. epidermidis* cultures treated with Cu-NPs and TiO_2_-NPs, resulting in a 2.2- and 13.7-fold increase in its content compared to the control sample. To conclude, the generation of ^•^OH was more affected by NPs in *S. epidermidis* cells than in *E. coli* and *B. cereus*.

Although ^1^O_2_ is one of the commonly studied ROS during photooxidative stress studies, it was important to distinguish whether ^1^O_2_ can be generated as a result of NPs action and modifications in redox reactions without prior irradiation. The results obtained for *E. coli* showed that only ZnO-NPs generated ^1^O_2_ in bacterial cells, causing about a 6-fold increase in its content compared to unstressed cells. Whilst treatment of *B. cereus* cells with Ag-NPs, Cu-NPs, ZnO-NPs and TiO_2_-NPs did not influence any significant (*p* > 0.05) alternations in ^1^O_2_ concentration. By contrast, the application of Ag-NPs and Cu-NPs to *S. epidermidis* cultures resulted in a greater, about 13.5- and 4.15-fold generation of ^1^O_2_ compared to the control sample, respectively (Figure 1C).

In parallel experiments, the ability of NPs to generate the O_2_^•−^ was evaluated (Figure 1D). The O_2_^•−^ is one of the crucial types of ROS to be widely examined because it is the first product of single-electron reduction of molecular oxygen and the substrate for SOD reaction, consequently increasing the content of H_2_O_2_. The obtained results revealed that Cu-NPs and ZnO-NPs had the greatest impact on the O_2_^•−^ production by *E. coli* compared to the non-treated cells. Interestingly, treatment of *E. coli* cells with Ag-NPs did not have a stimulating effect on the generation of O_2_^•−^. Exposure of *B. cereus* to ZnO-NPs resulted in an 18.4-fold higher level of O_2_^•−^ in the cells compared to the control sample. Intriguingly, both Ag-NPs and Cu-NPs resulted in a similar generation level of O_2_^•−^ in *B. cereus*. The obtained data for *S. epidermidis* showed that all tested NPs had a stimulating effect on the generation of O_2_^•−^, with the greatest ability shown by Cu-NPs and TiO_2_-NPs (14.5- and 23.4-fold increase, respectively). In general, the increase in O_2_^•−^ level in *S. epidermidis* was affected by the NPs in the following order Ag-NPs < ZnO-NPs < Cu-NPs < TiO_2_-NPs. In conclusion, the tested NPs generated the highest amount of O_2_^•−^ in *S. epidermidis*, while this process was less intense in *E. coli* and *B. cereus*. Additionally, based on the total concentration of ROS in bacterial cells (Figure 1E), it can be concluded that Cu-NPs generated the major quantity of different types of ROS in *E. coli* and *B. cereus* cells. In turn, the metal oxides TiO_2_-NPs and ZnO-NPs had the greatest impact on ROS production in *S. epidermidis* cells (9.5- and 11.5-fold increase, respectively).

### 2.3. Activity of Antioxidant Enzymes and Dehydrogenases under NPs Stress

**Figure 2:**
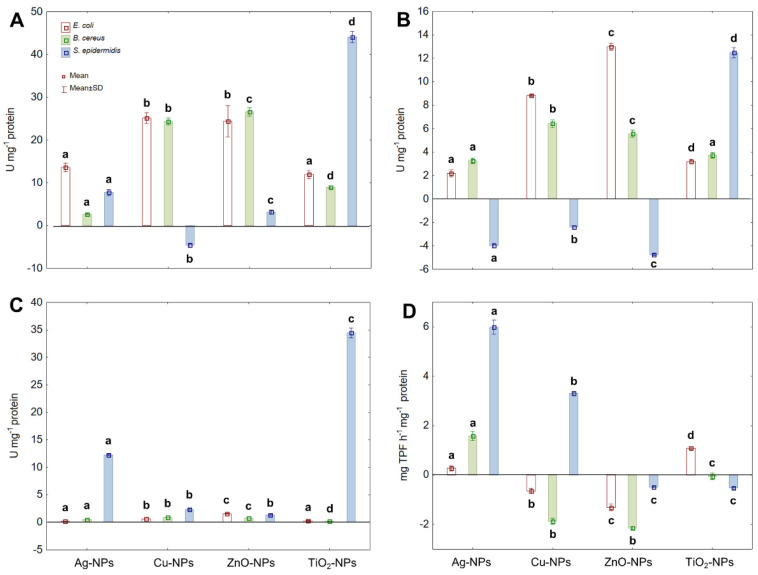
The activity of CAT (**A**), PER (**B**), SOD (**C**) and DEH (**D**) in *E. coli*, *B. cereus* and *S. epidermidis* exposed to NPs at an IC_50_ value (mean ± SD; *n* = 3). Means with the same letter(s) are not significant at *p* < 0.05 within each enzyme between the control and NPs-treated cells.

The ability of NPs to generate various types of ROS leads to oxidative stress in microbial cells and creates potential hazards for the survival of microorganisms. To assess the performance of the catalytic antioxidant defence system operating in NPs-treated bacterial cells, CAT, PER and SOD activities were measured. Additionally, the activity of DEH belonging to oxidoreductases was calculated to define the effect of NPs on microbial respiratory activity and their potential co-dependence in ROS generation. The obtained findings showed that all tested NPs caused an increase in the activity of CAT in *E. coli*, *B. cereus* and *S. epidermidis* (Figure 2A). Concerning *E. coli*, Ag-NPs and TiO_2_-NPs manifested a weak stimulating effect on CAT activity (about a 1.5-fold increase), while Cu-NPs and ZnO-NPs showed a stronger stimulating effect (over 2-fold increase). Similarly, Cu-NPs and ZnO-NPs stimulated CAT activity in *B. cereus*, causing a 6–7 fold increase compared to CAT activity in the untreated cells. Conversely, the greatest increase in CAT activity (about 4.8-fold) in *S. epidermidis* was recorded in the presence of TiO_2_-NPs. Interestingly, the exposure of *S. epidermidis* to Cu-NPs resulted in a noticeable decrease (about 1.53-fold) in the activity of CAT. Conclusively, CAT of *E. coli* and *B. cereus* was more sensitive to stress caused by tested NPs than CAT of *S. epidermidis*; however, the last bacterium was most sensitive to TiO_2_-NPs.

Parallelly, the activity of PER in tested bacteria under NPs-stress conditions was analysed (Figure 2B). During reactions catalysed by PER, H_2_O_2_ is reduced to water contributing to its overall concentration in a cell. The addition of Cu-NPs and ZnO-NPs to *E. coli* cultures substantially enhanced PER activity (2.2- and 2.8-fold, respectively), whereas Ag-NPs and TiO_2_-NPs had a smaller impact on its activity (about 1.4-fold increase). The treatment of *B. cereus* with Cu-NPs and ZnO-NPs also resulted in a noticeable increase in PER activity (about 7-fold) in comparison with its activity in the control samples. Conversely, exposure of *S. epidermidis* to Ag-NPs, Cu-NPs and ZnO-NP caused a 4.3-, 1.8- and 12.9-fold decrease in PER activity, respectively. The only type of NPs that acted in the opposite way to PER activity were TiO_2_-NPs causing a 3.8-fold increase compared to the untreated cells. It is noteworthy that PER functioning, however different in *E. coli* and *S. epidermidis* strains, was more sensitive to NPs-stress than PER of *B. cereus*, which in turn proved to be more resistant to NPs action.

The following assayed enzyme protecting microbial cells from oxidative stress was SOD, which catalyses the dismutation of O_2_^•−^ to O_2_ and H_2_O_2_ further used as a substrate in catalytic protection of bacterial cells against ROS. Although exposure of tested bacteria to NPs significantly affected SOD activity, it differed significantly between individual strains (Figure 2C). Detailed analysis of data showed that the highest increase in SOD activity in *E. coli* (1.6- and 2.6-fold) was recorded in the presence of Cu-NPs and ZnO-NPs, respectively. The same NPs in the culture of *B. cereus* also had the most stimulating effect on the activity of SOD, resulting in about a 1.5-fold increase in its activity. By comparison, *S. epidermidis* exhibited the highest SOD activity under Ag-NPs and TiO_2_-NPs treatments, achieving a 2.7- and 5.7-fold increase in its activity compared to the control cells.

Regarding DEH, its activity was expressed in a strain-specific manner under individual NPs treatment (Figure 2D). In *E. coli*, the displayed DEH activity was about 1-fold enhanced by Ag-NPs and TiO_2_-NPs; however, Cu-NPs and ZnO-NPs led to a significant decrease (1.3-fold) in its activity compared to the untreated cells. In turn, the DEH activity in *B. cereus* only increased after exposure to Ag-NPs, whereas Cu-NPs, ZnO-NPs and TiO_2_-NPs reduced its activity by 2.8-, 3.2- and 1.4-fold, respectively. By comparison, Ag-NPs and Cu-NPs strongly stimulated DEH activity in *S. epidermidis*, resulting in a 6.5- and 4-fold increase, respectively. Contrary, TiO_2_-NPs and ZnO-NPs caused a significant decrease (2.4-fold) in DEH activity in comparison with the control cells. The presented findings indicated that DEH was more affected by NPs in *B. cereus* and *S. epidermidis* than in *E. coli.*

### 2.4. Non-Catalytic Antioxidant Defence System (GSH)

**Figure 3:**
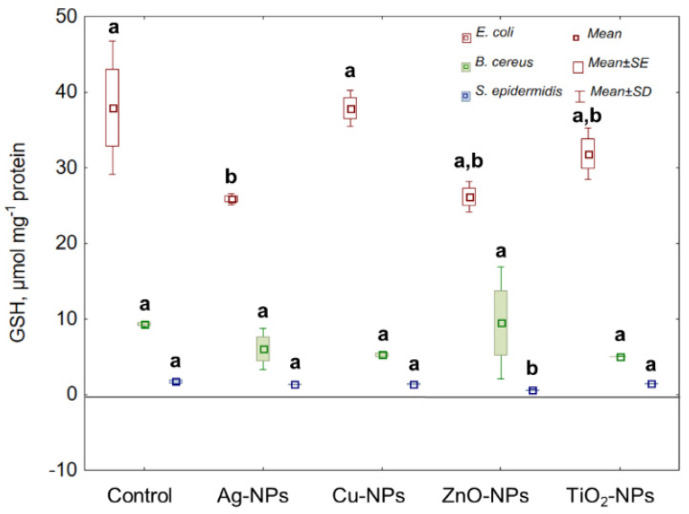
The GSH concentration in *E. coli*, *B. cereus* and *S. epidermidis* exposed to NPs at an IC_50_ value (mean ± SD/SE; *n* = 3). Means with the same letter(s) are not significant at *p* < 0.05 between the control and NPs-treated cells.

Reduced glutathione (GSH) acts as a hydrogen donor in the detoxification of H_2_O_2_; therefore, it plays an important role in a complex bacterial antioxidant defence system regulating the redox cycles. As Figure 3 indicates, the greatest reduction in the GSH level in *E. coli* was established under ZnO-NPs and Ag-NPs exposure, causing about a 1.5-fold decrease in its concentration compared to the control sample. Interestingly, no significant differences were found in the level of GSH in *B. cereus* cultured with individual NPs. Comparable to the reduction in GSH concentration in *E. coli* was the decrease (approximately 3-fold) in GSH in *S. epidermidis* treated with ZnO-NPs. Overall, ZnO-NPs were the only shared NPs for *E. coli* and *S. epidermidis*, inducing a significant reduction in their GSH levels.

### 2.5. Changes in the Content of Carbonyl and Amine Groups in Proteins

**Figure 4:**
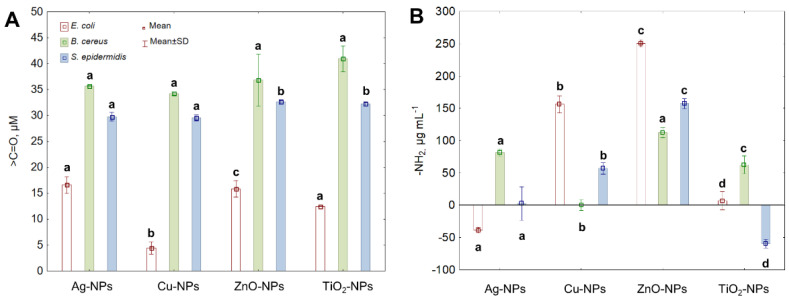
The content of >C=O (**A**) and -NH_2_ (**B**) in *E. coli*, *B. cereus* and *S. epidermidis* cells exposed to NPs at an IC_50_ value (mean ± SD/SE; *n* = 3). Means with the same letter(s) are not significant at *p* < 0.05 between the control and NPs-treated cells.

Interactions of NPs and ROS with proteins can cause various structural changes resulting from their oxidation and causing the disturbance of their function. One of the consequences of protein oxidation is the increase in the level of protein carbonyls (>C=O); therefore, it is recognized as a reliable biomarker of cellular changes induced by oxidative stress. As shown in Figure 4A, the exposure of *E. coli*, *B. cereus* and *S. epidermidis* to all tested NPs increased >C==O content. In *E. coli*, the greatest and a comparable increase in >C=O by 12.43%, 11.79% and 10.29% was recorded after treatment with Ag-NPs, ZnO-NPs and TiO_2_-NPs, respectively. By comparison, the highest >C=O content of about 31% in *B. cereus* was confirmed under TiO_2_-NPs and ZnO-NPs exposure. Similar findings were obtained for *S. epidermidis* exhibiting a significant increase in >C=O by 25.53% and 26.22% after treatment with TiO_2_-NPs and ZnO-NPs, respectively. In general, protein oxidation was a more predominant trait of NPs in *B. cereus* and *S. epidermidis* than in *E. coli*.

Protein oxidation can lead to irreversible changes in amine groups (-NH_2_), accompanied by protein aggregation and degradation. For this reason, it was essential also to distinguish the changes in -NH_2_ content in bacterial cells further to explore the oxidation effect of NPs and ROS. The highest increase in -NH_2_ by 45.08% in *E. coli* was detected in the presence of ZnO-NPs. Intriguingly, Ag-NPs caused a reduction in -NH_2_ content in *E. coli* by 5.42%. Correspondingly, the greatest increase in -NH_2_ by 18.52% and 23.03% in *B. cereus* and *S. epidermidis* was recorded in the presence of ZnO-NPs. Rivetingly, a decrease in -NH_2_ concentration by 8.39% in *S. epidermidis* compared to the control cells was detected after treatment with TiO_2_-NPs (Figure 4B).

### 2.6. Lipid Peroxidation in Bacterial Cells Caused by NPs

**Figure 5:**
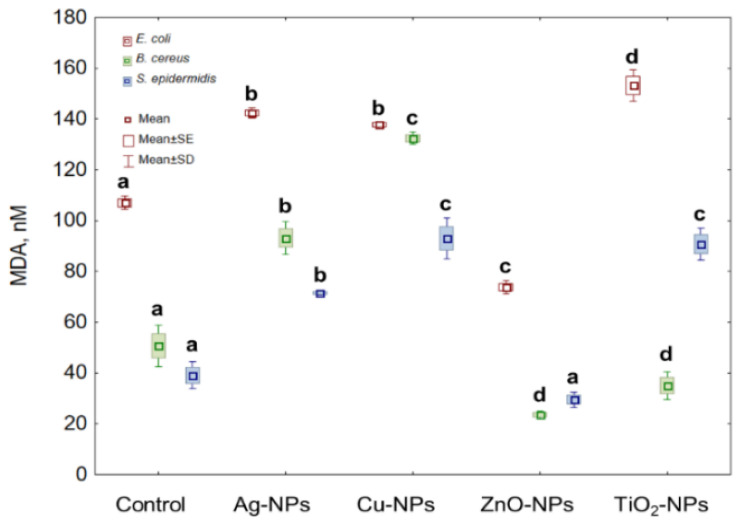
The LPO level in *E. coli*, *B. cereus* and *S. epidermidis* cells exposed to NPs at an IC_50_ value (mean ± SD/SE; *n* = 3). Means with the same letter(s) are not significant at *p* < 0.05 between the control and NPs-treated cells.

Lipid peroxidation (LPO) is one of the markers used in the analysis of oxidative stress. Therefore, this study seems necessary to distinguish the synergistic or antagonistic action between tested NPs and generated ROS. Treatment of *E. coli* cells with Ag-NPs, Cu-NPs and TiO_2_-NPs resulted in a significant increase in LPO level, while in the presence of ZnO-NPs the decrease in its content was observed. The highest and the lowest LPO values of 43.23% and 33.8% were obtained for the cells exposed to TiO_2_-NPs and ZnO-NPs, respectively. By comparison, the highest increase in the LPO values for *B. cereus* was documented at 160.95% for Cu-NPs treated cells compared to the LPO content in the untreated cells. Simultaneously, a 53.7% decrease in LPO was caused by ZnO-NPs in these cells. The obtained data also revealed the increase in LPO in *S. epidermidis* in the presence of Ag-NPs, Cu-NPs and TiO_2_-NPs and the decrease in the presence of ZnO-NPs. The exposure of *S. epidermidis* to Cu-NPs resulted in the highest increase in LPO values (194.49%), whereas the highest decrease (16.43%) occurred in the presence of ZnO-NPs (Figure 5).

### 2.7. Exploratory Data Analyses

**Figure 6:**
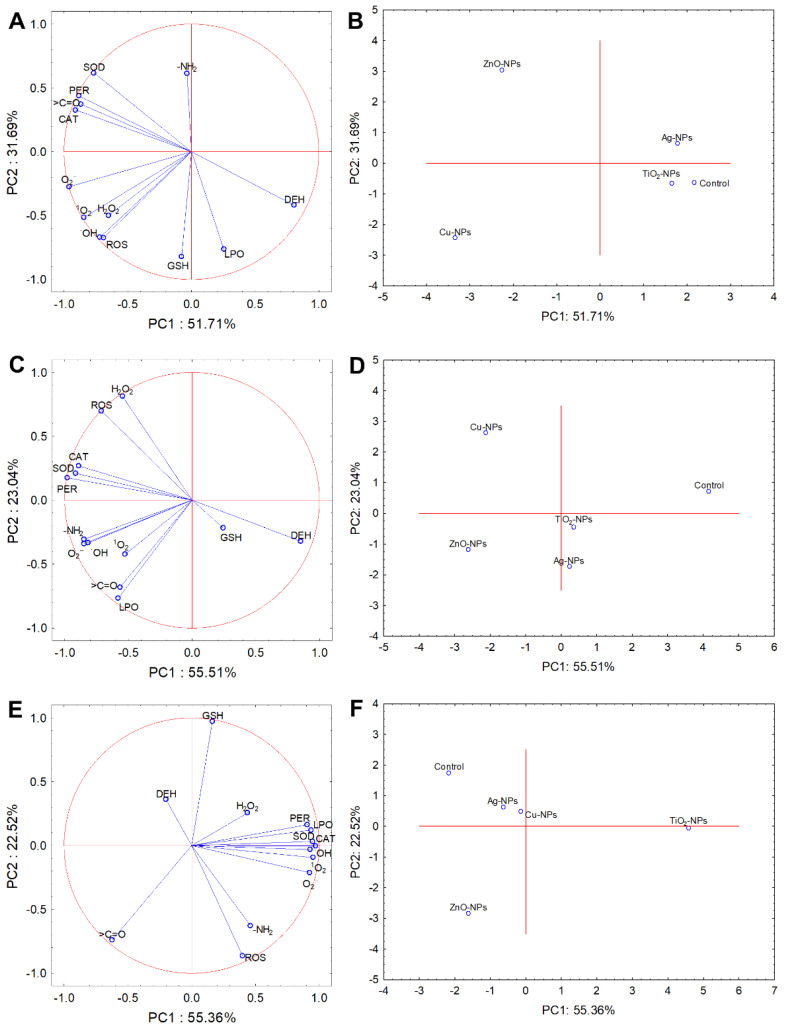
Projection of the individual plot and PCA analysis of free radicals (H_2_O_2_, ^•^OH, ^1^O_2_, O_2_^•−^), ROS, GSH, LPO, content of >C=O and -NH_2_, and enzymes activity (CAT, PER, SOD, DEH) along PC1 and PC2 for the control and NPs-treated cells of *E. coli* (**A**,**B**), *B. cereus* (**C**,**D**) and *S. epidermidis* (**E**,**F**).

PCA and cluster analysis were carried out to analyse the variability and relationship between obtained findings and performed experiments. The results from PCA, including all NPs treatments and toxicological studies performed for *E. coli*, *B. cereus* and *S. epidermidis* explained 83.40%, 78.55% and 77.88% variability of the data, respectively (Figure 6). Coordination biplot of experiments with *E. coli* demonstrated a strong negative correlation between CAT, PER and SOD activity and >C=O content (Figure 6A). Interestingly, a negative correlation between PER and DEH was established. The O_2_^•−^ was most correlated with PC1; however, based on PC2, a strong negative correlation was established for GSH and LPO, and a positive correlation for -NH_2_. Additionally, PC1 from the correlation biplot projection of *E. coli* distinguished three separate clusters of treated samples (Figure 6B). Based on PCA analysis, the results obtained for *E. coli* treated with Ag-NPs and TiO_2_-NPs were similar to the untreated samples forming one plot. In turn, coordination biplot for *B. cereus* revealed a strong negative correlation of SOD and PER, with other oxidative stress parameters being more scattered (Figure 6C). It is worth pointing out that a strong negative correlation was confirmed between ROS and GSH as well as DEH and CAT. Furthermore, based on the performed experiments, two clusters of treated samples of *B. cereus* along PC1 were distinguished: Ag-NPs with TiO_2_-NPs and Cu-NPs with ZnO-NPs, and an additional separate cluster consisting of the control sample (Figure 6D). Based on the PCA analysis for *S. epidermidis*, the most visible positive correlation with PC1 was observed for all parameters except for DEH, H_2_O_2_, GSH, -NH_2_, >C=O and ROS, which were strongly correlated with PC2. It is also worth emphasising the strong negative correlation between DEH and ROS (Figure 6E). Of all NPs tested, only TiO_2_-NPs were distinguished along the PC1 axis (Figure 6F).

**Figure 7:**
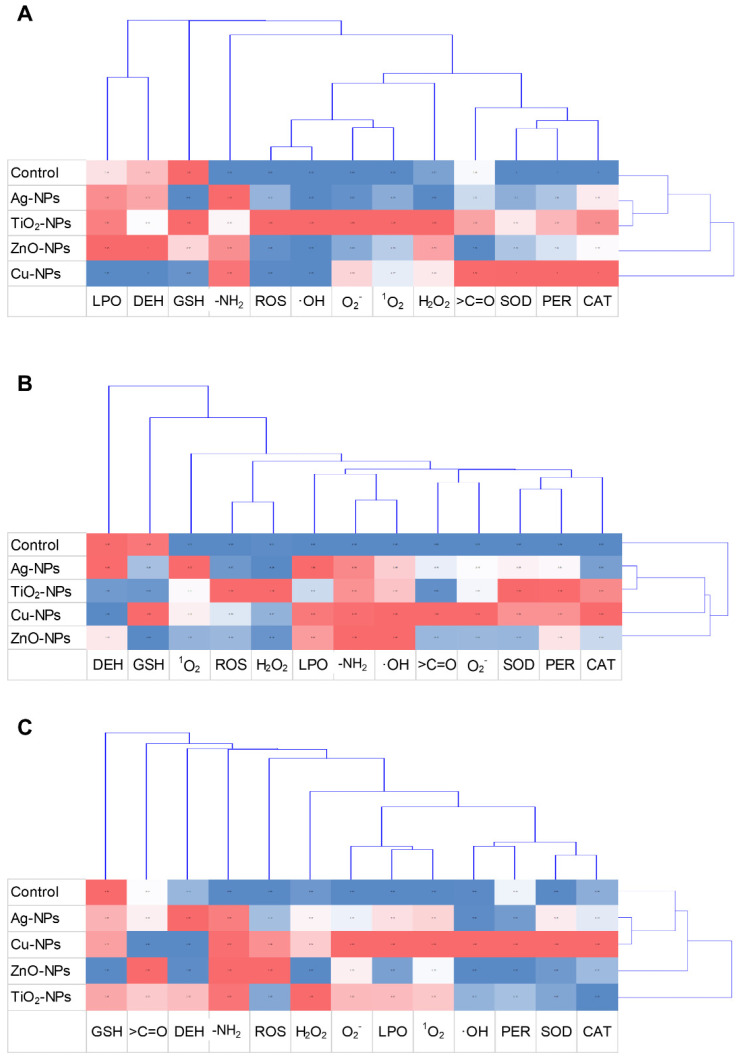
Cluster disposal of free radicals (H_2_O_2_, ^•^OH, ^1^O_2_, O_2_^•−^), total ROS, GSH, LPO, the content of >C=O and -NH_2_, and CAT, PER, SOD, DEH activities in the control and NPs-treated cells of *E. coli* (**A**), *B. cereus* (**B**) and *S. epidermidis* (**C**).

The cluster analysis showed characteristic relationships between particularly treated samples and conducted analysis (Figure 7). The dendrogram projection obtained for the *E. coli* strain revealed that the most differentiating analyses included measurement of LPO, DEH, GSH and protein oxidation expressed as -NH_2_ group level (Figure 7A). By comparison, other performed analyses, formed hierarchical clusters separating into two thematically similar clusters, including antioxidant enzymes and general and specific ROS levels. It is worth underlying that according to the acquired dendrogram, the most characteristic and discriminating NPs were Cu-NPs and ZnO-NPs. However, the most similar set of results were confirmed for Ag-NPs and TiO_2_-NPs. The dendrogram designated for *B. cereus* revelated two separate clusters, the first consisting of control samples and the second of specific NPs treatments (Figure 7B). It is worth pointing out that the most distinguished toxicological analyses with *B. cereus* included GSH, DEH and ^1^O_2_. Comparing, ROS, GSH, DEH and protein oxidation (-NH_2_ and >C=O levels) were the most differentiating analyses for *S. epidermidis* (Figure 7C). Dendrogram made for *S. epidermidis* demonstrated a strong relationship between Ag-NPs and Cu-NPs treated samples; however, exposure of these bacteria to ZnO-NPs and TiO_2_-NPs had the most divergent effect on the collected results.

## 3. Discussion

Incorporating nano-derived applications into daily use creates many solutions for the utility of different products and technological processes. Although the mathematical models based on predicted life cycles of various NPs estimate their possible unintentional targets sites and endpoints, there is still limited knowledge of how such materials will act in realistic conditions. One of the topics of the currently ongoing scientific debate on the toxicity of NPs is their potentially destructive effect on the functioning of microorganisms. Despite the huge amount of scientific data in this field, there is still controversy about the impact of NPs on microorganisms, especially these newly designed engineered NPs [7,8,24,25].

Among the several proposed mechanisms of action of metallic NPs on bacterial cells is the induction of oxidative stress by generating ROS [8,9,24,26]. Although this phenomenon has been reported in many publications, information on the individual radical forms induced by metal NPs and their combined action in disrupting cellular redox cycles remains fragmentary. Therefore, the novelty of this study is a scientific description of detailed oxidation profiles of *E. coli*, *B. cereus* and *S. epidermidis*, taking into account the accompanying pathological changes in the presence of redox unstable Ag-NPs, Cu-NPs, ZnO-NPs and TiO_2_-NPs.

To ensure the standardisation of nanotoxicological studies, it is important to investigate the potential toxicity of NPs by determining toxicological parameters. The conducted research confirmed the antimicrobial effect of all NPs on the tested bacterial strains, but to a different extent for individual strains. Overall, NPs were most potent against *E. coli* than *B. cereus* and *S. epidermidis.* This can be attributed to the difference in the structure of the outer layers of gram-negative and gram-positive bacteria. The cell wall of gram-negative bacteria has a thin layer of peptidoglycan, which makes it more permeable to macromolecules. Moreover, gram-negative bacteria are characterised by a higher net negative charge on the cell surface than gram-positive bacteria, which results in stronger electrostatic interactions with positively charged NPs and, therefore, the greater susceptibility of these microorganisms to the antimicrobial effect of NPs [9,24,27,28]. By comparison, *B. cereus* may be more resistant to NPs than *E. coli*, not only because of the thicker cell wall but also due to the formation of endospores that portray additional outer layers, thus providing additional protection against the negative effects of NPs [28,29]. Similar results were obtained by Ahmad et al. [28], who confirmed greater toxicity of TiO_2_-NPs towards *E. coli* than *B. subtilis* due to the early induction of cell death as opposed to the reduction of bacterial viability over time. Interestingly, *B. cereus* and *S. epidermidis* showed a similar response to the presence of tested NPs except for TiO_2_-NPs. This could be explained by the increased interaction of TiO_2_ with the functional groups on the surface of *B. cereus* cells. It can also result from the ability of *S. epidermis* to form a biofilm structure in the presence of TiO_2_-NPs and greater expression of cell wall anchored surface proteins [30]. Generally, *E. coli*, *B. cereus* and *S. epidermidis* were most susceptible to single metal NPs: Ag-NPs and Cu-NPs. This may be attributed to the faster release of heavy metal ions from their surface compared to metal oxide NPs. Furthermore, it is worth pointing out that the release of ions from NPs is dependent on their composition, physicochemical properties, culture medium and treated microorganisms; hence, there is no general rule for the dissolution of ions from different types of NPs [9,31,32,33]. Moreover, due to similar electron configuration and chemistry, Ag (I) and Cu (I) may exhibit similar antimicrobial properties; however, Cu plays an essential role in the physiological processes of microbial cells [9,34]. Therefore, depending on the concentration of cations released from the surface of NPs, microorganisms can or not withstand the stress caused by those ions through the activation of protective/resistance systems [9].

Treatment of *E. coli*, *B. cereus* and *S. epidermidis* with Ag-NPs, Cu-NPs, ZnO-NPs and TiO_2_-NPs induced ROS production in the cells. However, the total ROS concentration in *E. coli* and *B. cereus* was mostly influenced by Cu-NPs, while in *S. epidermidis* by ZnO-NPs and TiO_2_-NPs, which also influenced the production of particular types of ROS. The greatest contribution to the total ROS concentration in *E. coli* cells had H_2_O_2_ and ^•^OH, which levels increased in the presence of Cu-NPs. Similarly, there was a significant increase in the level of ^1^O_2_ in these cells after treatment with ZnO-NPs. Contrary, O_2_^•−^ and H_2_O_2_ had the greatest effect on overall ROS concentration in *B. cereus* exposed to ZnO-NPs and Cu-NPs, respectively. By comparison, all types of individual ROS slightly contributed to the general concentration of ROS in *S. epidermidis*. The positive correlation between the H_2_O_2_ and ^•^OH generation in *E. coli* can be related to the co-dependent existence of two types of ROS in bacterial cells. This is because hydrogen peroxide, a common-by product of the metabolic activity, undergoes Fenton and Fenton-like reactions generating ^•^OH, making its concentration in biological systems dependent on H_2_O_2_ [12,13,35]. Similarly, the level of H_2_O_2_ in cells depends on the production of O_2_^•−^; hence, there was a positive correlation between those two types of ROS in *B. cereus* [36]. Additionally, Ag-NPs, Cu-NPs, ZnO-NPs and TiO_2_-NPs can generate ^•^OH in bacterial cells through the release of transition metals that can participate in Fenton and Fenton-like reactions [9,37]. Here, both Cu-NPs and TiO_2_-NPs had the most stimulating effect on ROS formation in bacterial cells. Their smaller size than Ag-NPs and ZnO-NPs may explain the above-mentioned phenomenon. Smaller NPs compared to their larger counterparts are characterised by greater surface area to volume ratio, structural and electronic modifications, which provide more reactive sites and groups on the surfaces of NPs, that could participate in the generation of ROS [9,10]. Furthermore, the induction of ROS generation by Ag-NPs, Cu-NPs, ZnO-NPs and TiO_2_-NPs in bacterial cells could be caused by the suppression of the activity of respiratory enzymes through their interactions with released metal ions, activation of oxidases, and interactions with cellular components [38,39,40]. It can be concluded that the data presented here and the literature data indicate that ROS generation by NPs is very specific and difficult to predict the effect.

The relationship between ROS generation and the functioning of the antioxidant defence system is only partially presented in the available literature. In this work, ROS generation and oxidative stress induction were confirmed by disrupting enzymatic antioxidant activity. Activities of CAT, PER and SOD in *E. coli* cells were mostly affected by ZnO-NPs and correlated with the lowest overall ROS concentration. By comparison, CAT activity in *B. cereus* was most affected by ZnO-NPs, whereas Cu-NPs were mainly impacted on PER and SOD functioning. Interestingly, the collected results for PER activity in *B. cereus* are related to a high concentration of H_2_O_2_ after treatment with Cu-NPs. Activities of antioxidant enzymes in *S. epidermidis* were greatly affected by TiO_2_-NPs and positively correlated with high levels of H_2_O_2_ and O_2_^•−^. It is worth emphasising that Cu-NPs caused a significant decrease in CAT activity in this bacterium, whilst Ag-NPs, Cu-NPs and ZnO-NPs lowered the activity of PER. The observed reduction in enzyme activity may be associated with the release of ions from NPs and their interactions with bacterial proteins resulting in their suppression or denaturation [9,41,42]. Concluding, the diversified activity of the bacterial antioxidant system indicates different mechanisms of protection and adaptation of *E. coli*, *B. cereus* and *S. epidermidis* cells to stress conditions caused by particular NPs. Knowledge regarding the influence of NPs on antioxidant enzymes is limited to selected nanomaterials and microorganisms. For example, Liao et al. [42] found that exposure of *P. aeruginosa* to Ag-NPs caused an increase in the activity of CAT, PER and SOD, depending on the time and concentration of NPs. In other experiments, Yuan et al. [43] recorded a significant decrease in SOD and CAT activities in *P. aeruginosa* and *S. aureus* treated with Ag-NPs. Comparable results were obtained by Huang et al. [41], who documented an inhibitory effect of Ag-NPs on CAT, PER and SOD activities in *P. chrysosporium*, correlated with oxidative stress caused by high levels of ROS. By comparison, based on the obtained results in this study, *E. coli*, *B. cereus* and *S. epidermidis* had a more efficient response to the oxidative effects of NPs due to their stimulating effect on the activity of antioxidant enzymes.

It has been well documented that the interaction of NPs with bacterial outer layers can disrupt the cell wall and membrane integrity. Furthermore, the production of ROS, including O_2_^•−^ can cause additional damage through their interaction with functional groups presented on the microbial surfaces as well as their reactivity with chemical bonds in peptidoglycan layers. The damaging effect of both NPs and ROS on bacterial outer layers can lead to a disturbance in respiratory activity and ATP production [9,17,44]. Dehydrogenases play an essential role in microbial metabolic activity, especially in the respiratory metabolism of oxygen and electron cycling, during which trace amounts of different ROS may be produced [45,46]. Here, it was found that Ag-NPs, Cu-NPs, ZnO-NPs and TiO_2_-NPs had a specific nano-effect on DEH functioning. The investigation revealed that Cu-NPs and ZnO-NPs decreased DEH activity in *E. coli*, whilst TiO_2_-NPs increased their activity. In turn, Cu-NPs, ZnO-NPs and TiO_2_-NPs caused a significant decrease in DEH activity in *B. cereus*. In the case of *S. epidermidis*, Ag-NPs and Cu-NPs induced DEH activity, whereas the remaining tested NPs decreased their activity. Generalising, alterations in the DEH activity in *E. coli*, *B. cereus* and *S. epidermidis* after treatment with NPs indicate damage to the cell outer layers and disturbance in their respiratory activity. Greater sensitivity of DEH to metal-oxide NPs may be attributed to the larger release of metal ions and their enhanced interactions with bacterial surfaces [9,43,47]. Nano-toxicological studies related to DEH functioning concern both their leakage from the microbial cells and their overall activity. Kumar et al. [38] documented a greater DEH release (by 41% and 23%) from *E. coli* exposed to ZnO-NPs and TiO_2_-NPs due to loss of cell integrity. Similar findings were obtained by Korshed et al. [47] and El-Kaliuoby et al. [48], who confirmed a stimulated leakage of lactate dehydrogenases (LDH) from *E. coli* cells exposed to Ag-NPs and chitosan biopolymer NPs from *P. aeruginosa* and *S. aureus*, respectively. These studies indicate a targeted inactivation of metabolic activity of bacterial cells by a variety of different NPs.

Reduced glutathione plays an important role in the defence of bacterial cells, regulating the redox cycles of microbial cells. The measurement of GSH level is a valuable biomarker for determining the physiological status of a cell in various stress conditions, including oxidative stress [49,50]. A significant reduction in GSH level was observed in *E. coli* after exposure to Ag-NPs and ZnO-NPs and *S. epidermidis* treated with ZnO-NPs. GSH, apart from scavenging high levels of H_2_O_2_, can have a high affinity to released heavy metal ions from NPs, acting as a buffering agent against their excess concentration, which can further lead to the increase in its oxidised state (GSSG) [42,51]. Furthermore, NPs and released ions can interact with GSH thiol groups, depleting its concentration and reducing the defence mechanism of bacterial cells against generated ROS [9,23]. This finding and the potential agglomeration of NPs may explain the differences in obtained results. Additionally, each microorganism has a unique defence mechanism against oxidative stress, characterised by a different abundance of GSH and its divergent importance in protecting bacterial cells. For example, GSH is more abundant in gram-negative than gram-positive bacteria [52]. The results obtained in this work are in accordance with this statement because they clearly present a greater share of GSH in the protection of *E. coli* than *B. cereus* and *S. epidermidis*, producing a low concentration of GSH. Kumar et al. [38] found that GSH concentration in *E. coli* treated with ZnO-NPs and TiO_2_-NPs (80 μg mL^−1^) decreased by 53% and 60%, respectively. Referring to this study, an opposite trend was observed as a larger depletion of GSH by 15% in *E. coli* cells treated with ZnO-NPs than TiO_2_-NPs. This was correlated with small overall ROS concentration in *E. coli* cells detected after treatment with metal-oxide NPs, suggesting an active non-catalytic antioxidant defence system against the accumulation of intracellular ROS. In other studies, Korshed et al. [47] and Huang et al. [41] recorded a depletion of GSH in *E. coli* and *P. chrysosporium* treated with Ag-NPs, probably associated with detoxification of generated ROS. Similarly, Yuan et al. [43] reported a significant decrease in GSH levels by 80% and 70% in *P. aeruginosa* and *S. aureus* exposed to Ag-NPs, respectively. The lower bactericidal effect of Ag-NPs in this work may be related to the bigger size of commercial NPs and hence the smaller release of Ag^+^ ions.

Disruption of redox homeostasis by ROS, released metal ions and oxidative activity of NPs may lead to the oxidative damage of proteins resulting from changes in their carbonyl and amine content [53,54]. Oxidation of proteins can result in the formation of carbonyls on protein side chains containing amino acids such as lysine, arginine, cysteine, or proline [55,56,57,58]. The formation of protein carbonyls causes significant structural changes in proteins, disrupting their functioning or even leading to their degradation [59]. In this study, Ag-NPs, Cu-NPs, ZnO-NPs and TiO_2_-NPs caused significant alternations in >C=O level in *E. coli*, *B. cereus* and *S. epidermidis*. In general, the most visible changes were observed in the presence of ZnO-NPs and TiO_2_-NPs. This is in accordance with high LPO levels in these bacteria as well as significant changes in CAT, PER, SOD and DEH activities. Moreover, further oxidation of protein side chains containing amine groups can also result in considerable changes in their overall content because these groups amongst sulphur-containing ones are the most susceptible to oxidative changes forced by ROS [58,60]. Here, it was also established that ZnO-NPs was characterised by the strongest stimulating effect on -NH_2_ content in bacterial cells. The knowledge concerning the impact of NPs on the content of carbonyl and amine groups in bacteria is very limited [53,54]; therefore, the obtained results can be considered innovative.

The formation of ROS, especially ^•^OH and ^1^O_2_ can initiate lipid peroxidation, which is an oxidation process of unsaturated fatty acids and other lipids leading to severe structural changes in bacterial outer layers, including changes in its permeability, fluidity, and damage to the building components [53,61,62]. Herein, it was revealed that Ag-NPs, Cu-NPs and TiO_2_-NPs caused a significant increase in LPO level in *E. coli* and *S. epidermidis*; however, in *B. cereus* a visible increase in this parameter occurred only after exposure to Ag-NPs and Cu-NPs. The collected data further proved a strong oxidative stress generation by tested NPs in all tested strains. Interestingly, the addition of ZnO-NPs to the culture medium caused a decrease in LPO levels in *B. cereus* and *S. epidermidis*, which may suggest an adaptation of these bacteria to oxidative stress. In a similar study, Chatterjee et al. [53] documented a 35- and 50-fold increase in lipid peroxidation level in *E. coli* cells in the presence of Cu-NPs in concentrations of 3.0 and 7.5 μg mL^−1^, respectively. Analogously, Quinteros et al. [19] observed a 60% increase in the oxidation of lipids and proteins in *E. coli* after exposure to Ag-NPs. An increase in malondialdehyde (MDA) content in *E. coli* treated with TiO_2_-NPs was also positively correlated with ROS production [63]. In another study, Kumar et al. [38] found a dose-depended increase in the formation of hydroperoxide ions and MDA levels in *E. coli* exposed to ZnO-NPs and TiO_2_-NPs, suggesting an increase in the lipid peroxidation. Those results indicated membrane damage in *E. coli*, further supported by an increased LDH release after treatment with NPs. Comparable results were obtained by Jain et al. [64], who recorded a greater MDA production in *E. coli* and *P. putida* in comparison with *B. cereus* and *S. aureus* strains. This may be attributed to greater activity of the *E. coli* enzymatic antioxidant system and additional protection of cells by GSH, which may prevent an increased production of MDA compared to *B. cereus* and *S. epidermidis* cells.

## 4. Materials and Methods

### 4.1. Bacterial Strains and Nanoparticles

In this study, three bacterial strains were tested for various responses to the exposure of selected metallic nanoparticles. They included gram-negative *Escherichia coli* (ATCC^®^ 25922™) and gram-positive *Bacillus cereus* (ATCC^®^ 11778™), and *Staphylococcus epidermidis* (ATCC^®^ 12228™) strains purchased from the American Type Culture Collection (ATCC). *E. coli* was maintained using Bacto™ Tryptic Soy Broth (cat. 211825; pancreatic digest of casein 17.0 g L^−1^, papaic digest of soybean 3.0 g L^−1^, dextrose 2.5 g L^−1^, sodium chloride 5.0 g L^−1^, dipotassium phosphate g L^−1^); however, *B. cereus* and *S. epidermidis* were passaged in Difco™ Nutrient Broth (cat. 234000; beef extract 3.0 g L^−1^, peptone 5.0 g L^−1^).

All these strains were exposed to four types of nanoparticles (NPs): Ag-NPs (cat. 576832), Cu-NPs (cat. 774081), ZnO-NPs (cat. 677450) obtained from Sigma-Aldrich company and TiO_2_-NPs (cat. US1019F) acquired from US Research. The size of Ag-NPs, Cu-NPs and ZnO-NPs ranged in <100 nm, 25 nm and <50 nm, respectively, while TiO_2_-NPs were 20 nm in size. All NPs were characterised by 97–99.5% purity. Before starting the actual experiment, the stock solutions of NPs in sterile Millipore Water were sonicated (Vibra-Cell™, 20 kHz) for 10–20 min to avoid their aggregation/agglomeration.

### 4.2. Experimental Design

**Figure 8:**
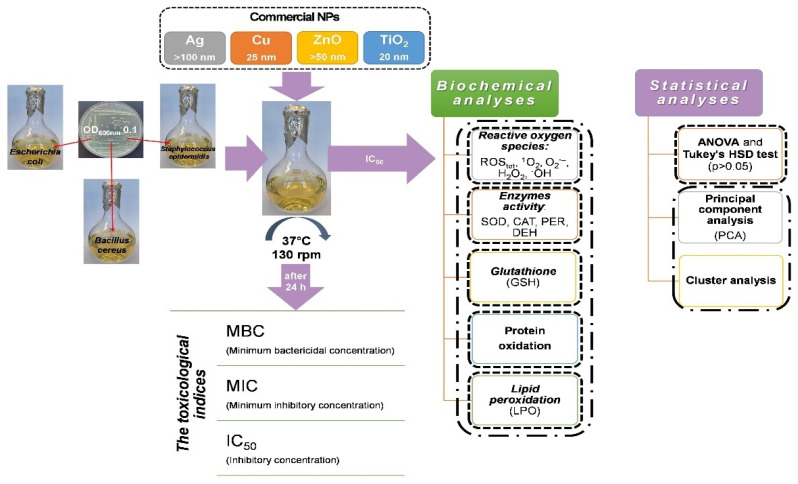
Scheme of experimental design.

To determine toxicological parameters of NPs and assess their influence on antioxidant defence system and other accompanying processes, bacteria were grown in lysogeny broth medium (LB mix; NaCl 10 g L^−1^, tryptone 10 g L^−1^, yeast extract 5 g L^−1^) until they reached half of the logarithmic growth phase (4–5 h). Subsequently, bacterial suspension in 0.85% NaCl containing cells from this phase was used to inoculate sterile LB medium until OD_600_ = 0.1 was achieved (~10^7^ CFU mL^−1^), and the appropriate NPs were individually added at a concentration corresponding to the IC_50_ values (Table 1). Depending on the assay performed, the bacterial cultures were incubated for 1 to 24 h at 37 °C. The experimental design with multifaceted analyses of measured parameters is presented in Figure 8.

### 4.3. Evaluating the Toxicological Effect of NPs on Bacterial Strains

The broth dilution method was performed to study the potential toxicological effect of individual Ag-NPs, Cu-NPs, ZnO-NPs and TiO_2_-NPs on bacterial cell viability [65,66]. The determined toxicological parameters included: minimum bactericidal concentration (MBC), minimum inhibitory concentration (MIC), and the half-maximal inhibitory concentration (IC_50_). Before bacteria treatment, serial-fold dilutions of NPs were prepared in a sterile LB medium with final concentrations ranging between 0 and 1200 mg L^−1^. Next, increasing concentrations of NPs were added to the bacterial cultures, which were incubated for 24 h at 37 °C with shaking (140 rpm). After that, 10-fold dilution series of each culture were prepared in 0.85% NaCl, and 100 μL of each bacterial suspension were sub-cultured on LB agar plates and incubated for 24 h at 37 °C. Afterwards, bacterial colonies were counted, and the number of bacteria was expressed as colony-forming units—CFU mL^−1^. Inhibition of bacterial growth was established using a mortality rate formula with 99% and 100% inhibition accounted for MIC and MBC values, respectively [65,66]. Accordingly, toxicological IC_50_ values of NPs were estimated with Prism 5 software (GraphPad Software, San Diego, CA, USA).

### 4.4. Measuring the Concentration of Reactive Oxygen Species (ROS)

The total concentration of ROS and levels of singlet oxygen (^1^O_2_), superoxide radical anion (O_2_^•−^), hydrogen peroxide (H_2_O_2_) and hydroxyl radical (^•^OH) were measured in biotic and abiotic samples treated with Ag-NPs, Cu-NPs, ZnO-NPs, and TiO_2_-NPs. Abiotic samples (research medium and NPs) were prepared to test the ability of NPs to spontaneously generate ROS, while the actual effect of NPs on ROS production in bacterial cells was assessed in the biotic trials. The final data concerning ROS production in the biotic samples are presented as the difference between biotic and abiotic samples for each NPs treatment. The total intracellular ROS concentration was evaluated using 2′,7′-dichlorodihydrofluorescein diacetate (H_2_DCFDA) oxidised to 2′,7′-dichlorofluorescein (DCF) by ROS [67]. First, bacterial cells from half of the log phase were suspended in phosphate-buffered saline (PBS) supplemented with individual NPs (IC_50_), and next, they were incubated for 1 h at 37 °C on a rotary shaker (140 rpm). Similarly, the appropriate blank samples were prepared without bacterial cells. After adding 4 mM H_2_DCFDA, the samples were incubated for 30 min, and the fluorescence was measured at an excitation wavelength of 485 nm and an emission wavelength of 530 nm. Meanwhile, the tested samples were cultured on LB agar plates, and CFU was calculated after 24 h of incubation at 37 °C. The intracellular ROS concentration was calculated according to Alpaslan et al. [68] and expressed as AU·CFU mL^−1^, where AU means absorbance unit.

The level of ^1^O_2_ was measured using a modified method by Zhang et al. [69] based on quenching of the 1,3-diphenylisobenzofuran (DPBF) fluorescence. The fluorescence of the abiotic and biotic samples prepared in PBS with NPs (IC_50_) was measured after the addition of 3 mM DBPF at λ = 410 nm over a 5 min period. Unlike, the O_2_^•−^ level was estimated in compliance with a method by Horst et al. [70], involving the reduction of XTT tetrazolium salt to a colourful formazan. After the addition of 91.3 mg L^−1^ XTT salt to the samples, the absorbance at λ = 490 nm was measured at 0 h and after 2 h of incubation with the reagent. Between the measurements, the samples were kept in the dark at 37 °C. The ^1^O_2_ and O_2_^•−^ level was expressed as AU. By comparison, H_2_O_2_ level was identified through the use of Amplex Red (AR) reagent and horseradish peroxidase (HRP), which converts AR to fluorescent resorufin in the presence of H_2_O_2_ measured at an excitation wavelength of 520 nm and an emission wavelength of 620 nm [71]. To measure the concentration of H_2_O_2_, 200 μM of AR reagent and 0.02 mg mL^−1^ of HRP were added to the samples, and the fluorescence was detected at 0 and next after 5, 10, 15 and 20 min of incubation. The concentration of H_2_O_2_ (μM) was estimated using a standard curve (y = 1592.9x) performed with concentrations ranging from 0.024 μM to 50 μM. The ^•^OH content was established using the indirect method of deoxyribose degradation, which leads to the formation of degradation products reacting readily with thiobarbituric acid (TBA) measured spectrophotometrically at λ = 532 nm [72,73]. The cells in PBS with NPs (IC_50_) and 20 mmol L^−1^ deoxyribose were incubated for 1 h at 37 °C and centrifuged (5000 rpm, 5 min, 4 °C). Next, 2.8% (*m*/*v*) trichloroacetic acid (TCA) and 1% (*w*/*v*) TBA were introduced to the supernatant and incubated for 15–20 min at 100 °C. After incubation, the absorbance of the sample was measured, and the concentration of ^•^OH was calculated using a standard curve (y = 8.832x) performed with malondialdehyde (MDA) concentrations ranging from 0 μM to 0.1 μM.

### 4.5. Measuring the Activity of Antioxidant Enzymes and Dehydrogenases

After exposure to NPs, the activities of three antioxidant enzymes: superoxide dismutase (SOD), catalase (CAT) and peroxidase (PER) in *E. coli*, *B. cereus* and *S. epidermidis* were assayed. The isolation of enzymes from bacterial cells was carried out in accordance with a method by Hegeman [74]. Bacteria were cultivated in LB medium with Ag-NPs, Cu-NPs, TiO_2_-NPs and ZnO-NPs (IC_50_) for 24 h at 37 °C and next, they were centrifuged (5000 rpm, 20 min, 4 °C). The resulting pellet was washed and suspended in 50 mM phosphate buffer (pH 7.0), and sonicated 6 times for 15 sec with 30-sec intervals (Vibra Cell™, 20 kHz) in the ice bath. The suspension was centrifuged (15,000 rpm, 20 min, 4 °C) and the obtained supernatant was subjected to the determination of the activity of enzymes. The activity of SOD was measured through an indirect method using a commercial reagent kit (cat. 19160; Sigma-Aldrich, St. Louis, MI, USA) based on the production of water-soluble colourful formazan dye and decrease in the colour intensity of the sample at λ = 450 nm. The specific activity of SOD was calculated using the formula described in the protocol by Zhang et al. [75]. The CAT activity was measured according to Banerjee et al. [76] and David et al. [77], based on the decomposition of H_2_O_2_ in time. A decrease in the absorbance of the sample at λ = 240 nm was measured over 3 min, and specific CAT activity was determined with molar extinction coefficient ε = 36,000 dm^3^·mol^−1^·cm^−1^. The PER activity was assessed using colorimetric protocol by Sigma-Aldrich based on the purpurogallin production. An increase in the absorbance of the sample at λ = 420 nm was measured for 3 min. The total protein concentration in the sample was determined according to Bradford [78] with Coomassie Brilliant Blue G-250 reagent and lysozyme as a standard. The activity of SOD, CAT, and PER was expressed as U mg^−1^ protein. To determine dehydrogenases (DEH) activity in bacteria, the common colorimetric assay based on the reduction of 2,3,5-triphenyltetrazolium chloride (TTC) to the creaming red-coloured triphenylformazan (TPF) was applied [79]. The activity of DHA was expressed as mg TPF h^−1^ mg^−1^ protein.

### 4.6. Measuring the Reduced Glutathione Concentration (GSH)

Glutathione is an important non-enzymatic component of the antioxidant system. The principal of the assay is based on the reaction between the sulfhydryl group of GSH with 5,5′-dithiobis(2-nitrobenzoic acid) (DTNB), known as Ellman’s reagent, leading to the production of colourful 5-thio-2-nitrobenzoic acid (TNB) [38]. After adding 100% TCA to the bacterial cultures exposed to NPs (IC_50_) for 24 h, they were incubated for 10 min at 37 °C and centrifuged (10,000 rpm, 5 min, 4 °C). The samples were then neutralised with Tris-HCl buffer (pH 8.9), and 0.01% DTNB was added. After 15–20 min of incubation, the absorbance of the sample was measured at λ = 412 nm. The concentration of GSH was estimated using a standard curve obtained for the concentrations ranging from 0.049 to 100 μM.

### 4.7. Determining Carbonyl and Amine Group Content in Oxidatively Modified Proteins

Determining the carbonyl (>C=O) and amine group (-NH_2_) content in proteins allows confirming protein oxidation in bacterial cells treated with NPs (IC_50_). The principal of the >C=O content assay is based on the reaction of protein carbonyl groups with 2,4-dinitrophenylhydrazine (DNPH), resulting in the formation of 2,4-dinitrophenylhydrazones measured calorimetrically [53,80,81]. Subsequently, -NH_2_ content measurement is based on the reaction of fluorescamine with amine groups, which produces fluorescent product proportional to the amine content in a sample, whilst the unbound fluorescamine hydrolyses to non-fluorescent products [60,81]. The supernatant for analysis was obtained in a similar way to the procedure for isolating antioxidant enzymes. To determine >C=O content, the proteins in the supernatant were precipitated by adding 10% (*w*/*v*) TCA and centrifuged (12,000 rpm, 15 min, 4 °C). The obtained precipitate was treated with 0.2% (*m*/*v*) DNPH and the sample was incubated for 1 h at 37 °C in the darkness. Sequentially, the precipitate was dissolved in guanidine hydrochloride, incubated for 15 min, and centrifuged (5000 rpm, 2 min, 4 °C). The absorbance of the collected supernatant was measured at λ = 370 nm, and the content of carbonyl groups was calculated using the molar extinction coefficient ε = 21 L·mmol^−1^·cm^−1^ of the formed hydrazone. In turn, to determine -NH_2_ content in proteins, 0.03% fluorescamine solution was added to the sample in a 3:1 ratio and then vortexed. After 30 min incubation in the dark, the fluorescence of a sample was measured at an excitation wavelength of 390 nm and an emission wavelength of 465 nm. The concentration of -NH_2_ was estimated using a standard curve obtained for standardised albumin samples in concentrations ranging from 50 to 1000 μg·mL^−1^.

### 4.8. Assessing the Lipid Peroxidation Level in Bacterial Strains

In the process of lipid peroxidation, one of the most common products is malondialdehyde (MDA), which reacts with TBA generating spectrophotometrically measured chromophore [53,82]. The supernatant was obtained analogously to the antioxidant enzyme isolation. In the next stage, 15% (*w*/*v*) TCA and 0.37% (*w*/*v*) TBA were added to the supernatant in 1:1:1 ratio. Parallelly, the control sample containing sterile H_2_O, instead of TBA, was prepared. The mixtures were incubated for 10 min at 100 °C and centrifuged (5000 rpm, 20 min, 4 °C). The absorbance of the solution was measured at λ = 535 nm, and the concentration of MDA was calculated based on the molar extinction coefficient ε = 156 L·mmol^−1^·cm^−1^.

### 4.9. Statistical Analysis

The assays were performed in three repeats for each sample set, and the final results were presented as the mean ± the standard deviation (SD)/the standard error (SE). The statistical significance of data in tested samples was determined through one-way ANOVA, followed by Tukey’s Honest Significant Difference test (HSD). The substantial variations in obtained data are represented by annotated letters in the presented graphics for the *p* < 0.05 statistical significance threshold. All statistical tests were carried out using STATISTICA 13.1 software package (Dell Inc., Austin, TE, USA).

## 5. Conclusions

This study revealed strong antimicrobial properties of Ag-NPs, Cu-NPs, ZnO-NPs and TiO_2_-NPs against *E. coli*, *B. cereus* and *S. epidermidis* strains and proved their antimicrobial effect depending on the species of bacteria and studied NPs. All NPs generated oxidative stress in the bacteria, which reflected in the production of different types of ROS in a wide range of concentrations. Disruption of bacterial redox homeostasis caused alterations in the activity of CAT, PER and SOD, depletion in GSH concentration and protein oxidation. Moreover, the research provided new insight into NPs effect on bacterial outer layers and respiratory metabolism through increased lipid peroxidation levels and alterations in DEH activity. Our ongoing research is focused on the expression of genes responsible for oxidative stress, redox homeostasis and antioxidant defence system, which will provide extra information on the genetic regulation of studied phenomena.

## Figures and Tables

**Table 1:** The values of MBC, MIC and IC_50_ (mg L^−1^) of NPs against *E. coli*, *B. cereus* and *S. epidermidis*.

Type of NPs	MBC	MIC	IC_50_
***Escherichia coli* ATCC 25922**			
Ag-NPs	15	10	7.84
Cu-NPs	250	200	180.80
ZnO-NPs	500	425	176.10
TiO_2_-NPs	750	500	43.40
***Bacillus cereus* ATCC 11778**			
Ag-NPs	1000	850	480.10
Cu-NPs	150	75	52.15
ZnO-NPs	1000	800	319.10
TiO_2_-NPs	150	100	50.30
***Staphylococcus epidermidis* ATCC 12228**			
Ag-NPs	600	500	442.20
Cu-NPs	300	200	112.00
ZnO-NPs	800	750	201.70
TiO_2_-NPs	>1200	1050	703.40

## Data Availability

All data that support the findings of this study are available from the corresponding authors upon reasonable request.

## References

[1] Qayyum S., Khan A.U. (2016). Nanoparticles vs. biofilms: A battle against another paradigm of antibiotic resistance. Med. Chem. Commun..

[2] Behera N., Arakha M., Priyadarshinee M., Pattanayak B.S., Soren S., Jha S., Mallick B.C. (2019). Oxidative stress generated at nickel oxide nanoparticle interface results in bacterial membrane damage leading to cell death. RSC Adv..

[3] Ameen F., Alsamhary K., Alabdullatif J.A., AlNadhari S. (2021). A review on metal-based nanoparticles and their toxicity to beneficial soil bacteria and fungi. Ecotoxicol. Environ. Saf..

[4] Liu J., Liu J., Attarilar S., Wang C., Tamaddon M., Yang C., Xie K., Yao J., Wang L., Liu C. (2020). Nano-modified titanium implant materials: A way toward improved antibacterial properties. Front. Bioeng. Biotechnol..

[5] Wang N., Fuh J.Y.H., Dheen S.T., Senthil Kumar A. (2021). Functions and applications of metallic and metallic oxide nanoparticles in orthopedic implants and scaffolds. J. Biomed. Mater. Res..

[6] Vergara-Llanos D., Koning T., Pavicic M.F., Bello-Toledo H., Díaz-Gómez A., Jaramillo A., Melendrez-Castro M., Ehrenfeld P., Sánchez-Sanhueza G. (2021). Antibacterial and cytotoxic evaluation of copper and zinc oxide nanoparticles as a potential disinfectant material of connections in implant provisional abutments: An in-vitro study. Arch. Oral Biol..

[7] Bundschuh M., Filser J., Lüderwald S., McKee M.S., Metreveli G., Schaumann G.E., Schulz R., Wagner S. (2018). Nanoparticles in the environment: Where do we come from, where do we go to?. Environ. Sci. Eur..

[8] Wang L., Hu C., Shao L. (2017). The antimicrobial activity of nanoparticles: Present situation and prospects for the future. Int. J. Nanomed..

[9] Slavin Y.N., Asnis J., Häfeli U.O., Bach H. (2017). Metal nanoparticles: Understanding the mechanisms behind antibacterial activity. J. Nanobiotechnol..

[10] Dayem A.A., Hossain M.K., Lee S.B., Kim K., Saha S.K., Yang G.M., Choi H.Y., Cho S.G. (2017). The role of reactive oxygen species (ROS) in the biological activities of metallic nanoparticles. Int. J. Mol. Sci..

[11] Canaparo R., Foglietta F., Limongi T., Serpe L. (2020). Biomedical applications of reactive oxygen species generation by metal nanoparticles. Materials.

[12] Imlay J.A. (2003). Pathways of oxidative damage. Annu. Rev. Microbiol..

[13] Collin F. (2019). Chemical basis of reactive oxygen species reactivity and involvement in neurodegenerative diseases. Int. J. Mol. Sci..

[14] Onyango A.N. (2016). Endogenous generation of singlet oxygen and ozone in human and animal tissues: Mechanisms, biological significance, and influence of dietary components. Oxidant Med. Cell Longev..

[15] Hubenko K., Yefimova S., Tkacheva T., Maksimchuk P., Borovoy I., Klochkov V., Kavok N., Opolonin O., Malyukin Y. (2018). 2018. Reactive oxygen species generation in aqueous solutions containing GdVO_4_:Eu^3+^ nanoparticles and their complexes with methylene blue. Nanoscale Res. Lett..

[16] Wang D., Zhao L., Ma H., Zhang H., Guo L.H. (2017). Quantitative analysis of reactive oxygen species photogenerated on metal oxide nanoparticles and their bacteria toxicity: The role of superoxide radicals. Environ. Sci. Technol..

[17] Muñiz Diaz R., Cardoso-Avila P.E., Pérez Tavares J.A., Patakfalvi R., Villa Cruz V., Pérez Ladrón de Guevara H., Gutiérrez Coronado O., Arteaga Garibay R.I., Saavedra Arroyo Q.E., Marañón-Ruiz V.F. (2021). Two-step triethylamine-based synthesis of MgO nanoparticles and their antibacterial effect against pathogenic bacteria. Nanomaterials.

[18] Li Y., Zhang W., Niu J., Chen Y. (2012). Mechanism of photogenerated reactive oxygen species and correlation with the antibacterial properties of engineered metal-oxide nanoparticles. ACS Nano.

[19] Quinteros M.A., Aristizábal V.C., Dalmasso P.R., Paraje M.G., Páez P.L. (2016). Oxidative stress generation of silver nanoparticles in three bacterial genera and its relationship with the antimicrobial activity. Toxicol. In Vitro.

[20] Makhdoumi P., Karimi H., Khazaei M. (2020). Review on metal-based nanoparticles: Role of reactive oxygen species in renal toxicity. Chem. Res. Toxicol..

[21] Fang F.C. (2011). Antimicrobial actions of reactive oxygen species. MBio.

[22] Ezraty B., Gennaris A., Barras F., Collet J.F. (2017). Oxidative stress, protein damage and repair in bacteria. Nat. Rev. Microbiol..

[23] Choi Y., Kim H.A., Kim K.W., Lee B.T. (2018). Comparative toxicity of silver nanoparticles and silver ions to *Escherichia coli*. J. Environ. Sci..

[24] Yang X.Y., Chung E., Johnston I., Ren G., Cheong Y.K. (2021). Exploitation of antimicrobial nanoparticles and their applications in biomedical engineering. Appl. Sci..

[25] Zhao J., Lin M., Wang Z., Cao X., Xing B. (2021). Engineered nanomaterials in the environment: Are they safe?. Crit. Rev. Environ. Sci. Technol..

[26] Baptista P.V., McCusker M.P., Carvalho A., Ferreira D.A., Mohan N.M., Martins M., Fernandes A.R. (2018). Nano-strategies to fight multidrug resistant bacteria—A battle of the titans. Front. Microbiol..

[27] Kubo A.L., Capjak I., Vrček I.V., Bondarenko O.M., Kurvet I., Vija H., Ivask A., Kasemets K., Kahru A. (2018). Antimicrobial potency of differently coated 10 and 50 nm silver nanoparticles against clinically relevant bacteria *Escherichia coli* and *Staphylococcus aureus*. Coloids Surf. B.

[28] Ahmad N.S., Abdullah N., Yasin F.M. (2020). Toxicity assessment of reduced graphene oxide and titanium dioxide nanomaterials on gram-positive and gram-negative bacteria under normal laboratory lighting condition. Toxicol. Rep..

[29] Bottone E.J. (2010). *Bacillus cereus*, a volatile human pathogen. Clin. Microbiol. Rev..

[30] Foster T.J. (2020). Surface proteins of *Staphylococcus epidermidis*. Front. Microbiol..

[31] Odzak N., Kistler D., Behra R., Sigg L. (2014). Dissolution of metal and metal oxide nanoparticles in aqueous media. Environ. Pollut..

[32] Wang D., Lin Z., Wang T., Yao Z., Qin M., Zheng S., Lu W. (2016). Where does the toxicity of metal oxide nanoparticles come from: The nanoparticles, the ions, or a combination of both?. J. Hazard. Mater..

[33] Ghorbani R., Biparva P., Moradian F. (2020). Assessment of antibacterial activity and the effect of copper and iron zerovalent nanoparticles on gene expression DnaK in *Pseudomonas aeruginosa*. BioNanoScience.

[34] Eckhardt S., Brunetto P.S., Gagnon J., Priebe M., Giese B., Fromm K.M. (2013). Nanobio silver: Its interactions with peptides and bacteria, and its uses in medicine. Chem. Rev..

[35] Fasnacht M., Polacek N. (2021). Oxidative stress in bacteria and the central dogma of molecular biology. Front. Mol. Biosci..

[36] Bond R.J., Hansel C.M., Voelker B.M. (2020). Heterotrophic bacteria exhibit a wide range of rates of extracellular production and decay of hydrogen peroxide. Front. Mar. Sci..

[37] Hong R., Kang T.Y., Michels C.A., Gadura N. (2012). Membrane lipid peroxidation in copper alloy-mediated contact killing of *Escherichia coli*. Appl. Environ. Microbiol..

[38] Kumar A., Pandey A.K., Singh S.S., Shanker R., Dhawan A. (2011). Engineered ZnO and TiO_2_ nanoparticles induce oxidative stress and DNA damage leading to reduced viability of *Escherichia coli*. Free Radic. Biol. Med..

[39] Manke A., Wang L., Rojanasakul Y. (2013). Mechanisms of nanoparticle-induced oxidative stress and toxicity. Biomed. Res. Int..

[40] Dwivedi S., Wahab R., Khan F., Mishra Y.K., Musarrat J., Al-Khedhairy A.A. (2014). Reactive oxygen species mediated bacterial biofilm inhibition via zinc oxide nanoparticles and their statistical determination. PLoS ONE.

[41] Huang Z., He K., Song Z., Zeng G., Chen A., Yuan L., Li H., Hu L., Guo Z., Chen G. (2018). Antioxidative response of *Phanerochaete chrysosporium* against silver nanoparticle- induced toxicity and its potential mechanism. Chemosphere.

[42] Liao S., Zhang Y., Pan X., Zhu F., Jiang C., Liu Q., Cheng Z., Dai G., Wu G., Wang L. (2019). Antibacterial activity and mechanism of silver nanoparticles against multidrug-resistant *Pseudomonas aeruginosa*. Int. J. Nanomed..

[43] Yuan Y.G., Peng Q.L., Gurunathan S. (2017). Effects of silver nanoparticles on multiple drug- resistant strains of *Staphylococcus aureus* and *Pseudomonas aeruginosa* from mastitis-infected goats: An alternative approach for antimicrobial therapy. Int. J. Mol. Sci..

[44] Chowdhuri A.R., Tripathy S., Chandra S., Roy S., Sahu S.K. (2015). A ZnO decorated chitosan–graphene oxide nanocomposite shows significantly enhanced antimicrobial activity with ROS generation. RSC Adv..

[45] Heikal A., Nakatani Y., Dunn E., Weimar M.R., Day C.L., Baker E.N., Lott J.S., Sazanov L.A., Cook G.M. (2014). Structure of the bacterial type II NADH dehydrogenase: A monotopic membrane protein with an essential role in energy generation. Mol. Microbiol..

[46] Billenkamp F., Peng T., Berghoff B.A., Klug G. (2015). A cluster of four homologous small RNAs modulates C1 metabolism and the pyruvate dehydrogenase complex in *Rhodobacter sphaeroides* under various stress conditions. J. Bacteriol..

[47] Korshed P., Li L., Liu Z., Wang T. (2016). The molecular mechanisms of the antibacterial effect of picosecond laser generated silver nanoparticles and their toxicity to human cells. PLoS ONE.

[48] El-Kaliuoby M.I., Amer M., Shehata N. (2021). Enhancement of nano-biopolymer antibacterial activity by pulsed electric field. Polymers.

[49] Masip L., Veeravalli K., Georgiou G. (2006). The many faces of glutathione in bacteria. Antioxid. Redox Signal..

[50] Smirnova G., Muzyka N., Oktyabrsky O. (2012). Transmembrane glutathione cycling in growing *Escherichia coli* cells. Mircobiol. Res..

[51] Stewart L.J., Ong C.L.Y., Zhang M.M., Brouwer S., McIntrye L., Davies M.R., Walker M.J., McEwan A.G., Waldron K.J., Djoko K.Y. (2020). A role for glutathione in buffering excess intracellular copper in *Streptococcus pyogenes*. MBio.

[52] Smirnova G.V., Oktyabrsky O.N. (2005). Glutathione in bacteria. Biochemistry.

[53] Chatterjee A.K., Chakraborty R., Basu T. (2014). Mechanism of antibacterial activity of copper nanoparticles. Nanotechnology.

[54] Singh R., Cheng S., Singh S. (2020). Oxidative stress-mediated genotoxic effect of zinc oxide nanoparticles on *Deinococcus radiodurans*. 3 Biotech.

[55] Dalle-Donne I., Rossi R., Giustarini D., Milzani A., Colombo R. (2003). Protein carbonyl groups as biomarkers of oxidative stress. Clin. Chim. Acta.

[56] Suzuki Y.J., Carini M., Butterfield D.A. (2010). Protein carbonylation. Antioxid. Redox Signal..

[57] Nayak J., Jena S.R., Samanta L., Henkel R., Samanta L., Agarwal A. (2019). Oxidative stress and sperm dysfunction: An insight into dynamics of semen proteome. Oxidants, Antioxidants and Impact of the Oxidative Status in Male Reproduction.

[58] Xiong Y.L., Guo A. (2021). Animal and plant protein oxidation: Chemical and functional property significance. Foods.

[59] Catalán V., Frühbeck G., Gómez-Ambrosi J., Marti del Moral A., Aguilera García C.M. (2018). Inflammatory and oxidative stress markers in skeletal muscle of obese subjects. Obesity, Oxidative Stress and Dietary Antioxidants.

[60] Wang S., Zhou Q., Chen X., Luo R.H., Li Y., Liu X., Yang L.M., Zheng Y.T., Wang P. (2021). Modification of *N*-terminal α-amine of proteins via biomimetic *ortho*-quinone-mediated oxidation. Nat. Commun..

[61] Bhattacharya P., Dey A., Neogi S. (2021). An insight into the mechanism of antibacterial activity by magnesium oxide nanoparticles. J. Mater. Chem. B.

[62] Grotto D., Santa Maria L., Valentini J., Paniz C., Schmitt G., Garcia S.C., Pomblum V.J., Rocha J.B.T., Farina M. (2009). Importance of the lipid peroxidation biomarkers and methodological aspects for malondialdehyde quantification. Quim. Nova.

[63] Lin X., Li J., Ma S., Liu G., Yang K., Tong M., Lin D. (2014). Toxicity of TiO_2_ nanoparticles to *Escherichia coli*: Effects of particle size, crystal phase and water chemistry. PLoS ONE.

[64] Jain N., Bhargava A., Rathi M., Dilip R.V., Panwar J. (2015). Removal of protein capping enhances the antibacterial efficiency of biosynthesized silver nanoparticles. PLoS ONE.

[65] Wiegand I., Hilpert K., Hancock R.E.W. (2008). Agar and broth dilution methods to determine the minimal inhibitory concentration (MIC) of antimicrobial substances. Nat. Protoc..

[66] Bagchi B., Kar S., Dey S.K., Bhandary S., Roy D., Mukhopadhyay T.K., Das S., Nandy P. (2013). In situ synthesis and antibacterial activity of copper nanoparticle loaded natural montmorillonite clay based on contact inhibition and ion release. Colloids Surf. B Biointerfaces.

[67] Yang Y., Wang J., Xiu Z., Alvarez P.J.J. (2013). Impacts of silver nanoparticles on cellular and transcriptional activity of nitrogen-cycling bacteria. Environ. Toxicol. Chem..

[68] Alpaslan E., Geilich B.M., Yazici H., Webster T.J. (2017). pH-controlled cerium oxide nanoparticle inhibition of both gram-positive and gram-negative bacteria growth. Sci. Rep..

[69] Zhang Y., Huang P., Wang D., Chen J., Liu W., Hu P., Huang M., Chen X., Chen Z. (2018). Near-infrared-triggered antibacterial and antifungal photodynamic therapy based on lanthanide-doped upconversion nanoparticles. Nanoscale.

[70] Horst A.M., Vukanti R., Priester J.H., Holden P.A. (2013). An assessment of fluorescence- and absorbance-based assays to study metal-oxide nanoparticle ROS production and effects on bacterial membranes. Small.

[71] Seaver L.C., Imlay J.A. (2001). Alkyl hydroperoxide reductase is the primary scavenger of endogenous hydrogen peroxide in *Escherichia coli*. J. Bacteriol..

[72] Rice-Evans C.A., Diplock A.T., Symons M.C.R., Rice-Evans C.A., Diplock A.T., Symons M.C.R. (1991). The detection and characterization of free radical species. Laboratory Techniques in Biochemistry and Molecular Biology, Techniques in Free Radical Research.

[73] Meghana S., Kabra P., Chakraborty S., Padmavathy N. (2015). Understanding the pathway of antibacterial activity of copper oxide nanoparticles. RSC Adv..

[74] Hegeman G.D. (1966). Synthesis of the enzymes of the mandelate pathway by *Pseudomonas putida* I. Synthesis of enzymes by the wild type. J. Bacteriol..

[75] Zhang C., Bruins M.E., Yang Z.Q., Liu S.T., Rao P.F. (2016). A new formula to calculate activity of superoxide dismutase in indirect assays. Anal. Biochem..

[76] Banerjee G., Pandey S., Ray A.K., Kumar R. (2015). Bioremediation of heavy metals by a novel bacterial strain *Enterobacter cloacae* and its antioxidant enzyme activity, flocculant production and protein expression in presence of lead, cadmium and nickel. Water Air Soil Pollut..

[77] David M., Krishna P.M., Sangeetha J. (2016). Elucidation of impact of heavy metal pollution on soil bacterial growth and extracellular polymeric substances flexibility. 3 Biotech.

[78] Bradford M.M. (1976). A rapid and sensitive method for the quantitation of microgram quantities of protein utilizing the principle of protein-dye binding. Anal. Biochem..

[79] Nweke C.O., Alisi C.S., Okolo J.C., Nwanyanwu C.E. (2007). Toxicity of zinc to heterotrophic bacteria from a tropical river sediment. Appl. Ecol. Env. Res..

[80] Levine R.L., Garland D., Oliver C.N., Amici A., Climent I., Lenz A.G., Ahn B.W., Shaltiel S., Stadtman E.R. (1990). Determination of carbonyl content in oxidatively modified proteins. Methods Enzymol..

[81] Rice-Evans C.A., Diplock A.T., Symons M.C.R., Rice-Evans C.A., Diplock A.T., Symons M.C.R. (1991). Detection of protein structural modifications induced by free radicals. Laboratory Techniques in Biochemistry and Molecular Biology, Techniques in Free Radical Research.

[82] Rice-Evans C.A., Diplock A.T., Symons M.C.R., Rice-Evans C.A., Diplock A.T., Symons M.C.R. (1991). Investigation of the consequences of free radical attack on lipids. Laboratory Techniques in Biochemistry and Molecular Biology, Techniques in Free Radical Research.

